# Media Portrayals of Suicide

**DOI:** 10.1371/journal.pmed.1000051

**Published:** 2009-03-17

**Authors:** The PLoS Medicine Editors

## Abstract

Journalists have a tremendous opportunity, say the*PLoS Medicine* Editors, to educate the public about mental health problems and their treatment.

In a cohort study in this month's *PLoS Medicine*, Nav Kapur (University of Manchester, United Kingdom) and colleagues report that young men (24 years and under) who had left the UK Armed Forces were at higher risk of suicide than either young men in the general population or those still in active service [1]. The risk appeared to be greatest in the first two years after discharge, in those with a short length of service, and in those of lower rank. There was a low rate of contact with mental health specialists in the year before death—just 14% for those under 20 years and 20% for those under 24 years. This study has identified a vulnerable group and highlights the need for targeted intervention to save lives.

What kind of interventions might work? In an expert commentary on the new study, Jitender Sareen and Shay-Lee Belik (University of Manitoba, Canada) highlight one example of a program that was specifically targeted at an at-risk military population, and also consider more general public health approaches to suicide prevention [2]. The success story is a multilayered program initiated by the US Air Force in 1996 (http://afspp.afms.mil/), which includes educating personnel about suicide prevention, helping them to deal with their emotional reactions to traumatic incidents, and providing guidelines for commanders on how and when to use mental health services. A cohort study found that the program was associated with a 33% relative risk reduction in suicide (the 1990–1996 population was designated as the “unexposed” cohort and the population in 1997–2002 was considered to be the “exposed” cohort) [3].

The more general public health approaches, drawn from a recent systematic review of suicide prevention programs [4], include educating physicians to detect and treat depression, restricting access to lethal means, and the safe media reporting of suicides. Given that sensationalized media reports about suicide may increase the risk of copycat suicides [5,6], Sareen and Belik urge reporters not to overhype Kapur and colleagues' findings (it is important to note, they say, that the overall prevalence of suicide in those who left the Armed Forces was very low).

There are previous examples of dramatic media portrayals of suicide in soldiers. For example, in 1995 there was a stream of dramatic Canadian newspaper reports about military personnel who committed suicide during or after UN peacekeeping duties in Bosnia [7]. But a subsequent case control study found no increased suicide risk in peacekeepers as a whole (although it did find an increase in a subgroup of air force personnel) [7]. Sareen and Belik's advice to reporters prompted us to consider three questions ahead of publishing Kapur and colleagues' study—what types of media reports are most likely to have an effect upon suicidal behavior, what constitutes safe media reporting, and what can *PLoS Medicine* do to promote the responsible reporting of the study?

Reporters have a choice about how they frame stories about mental illness.

The available evidence, which some psychiatrists argue is epidemiologically weak [8], suggests that media portrayals are more likely to be associated with suicidal behavior when they are prominent on the page (e.g., large headlines, photos of the body), appear in multiple places (e.g., several TV networks and newspapers), report celebrity deaths, and are based on real rather than fictional (e.g., TV soap opera) suicides [5,9]. Two particular studies are often cited as evidence for a copycat effect [10,11]. A series of highly publicized media reports of subway suicides in Vienna, Austria was linked to a rise in subway suicides [10], and the suicide of a famous Canadian journalist, Gaëtan Girouard, was also associated with a rise in the suicide rate [11].

To reduce the risk of copycat suicides, several organizations—such as the US Centers for Disease Control and the Samaritans (a UK charity)—have issued guidelines to the media on safe reporting of suicides [12,13]. There is some evidence that these guidelines may help to reduce the suicide rate, though further research is needed to prove their effectiveness [5]. For example, in the wake of the rise in Viennese subway suicides, the Austrian Association for Suicide Prevention, Crisis Intervention, and Conflict Resolution produced media guidelines to encourage less sensationalized reporting, after which there was a decline in such suicides [10]. In early 2009 the Canadian Psychiatric Association issued a position paper on safe media reporting [14]. The paper urges reporters to avoid the following: giving details of the suicide method; using “suicide” in the headline; including photos or admiration of the deceased; repetitive, excessive, or front page coverage; “exciting” reporting; romanticized or simplistic reasons for the suicide; the idea that suicide is unexplainable; and approval of the suicide.

The position paper also makes the crucial point that journalists have a tremendous opportunity to educate the public about mental health problems and their treatment. For example, media reports could remind readers that suicide usually results from a treatable mental illness and could include information on how to seek help for suicidal thoughts. Keith Hawton and Kathryn Williams from the University of Oxford in the UK have suggested evaluating “whether media portrayal of positive coping with adversity in circumstances that might have led to suicidal acts could provide a model that might also reduce suicidal behaviour” [5].

Returning to Kapur and colleagues' new study in *PLoS Medicine*, as editors we discussed the issue of suicide contagion with the corresponding author, Nav Kapur, ahead of publication. He felt that since this is a large-scale epidemiological study (rather than, say, a single case of a celebrity suicide), it would be very unlikely that media reports about the study could have a copycat effect. Based on previous examples of media reporting on suicide in the military [7], we did think there might be a risk of sensationalized media portrayals of the new study, so we took care to write a sober press release, adopting principles that we laid out in a previous editorial [15]. In the press release we included links to guidelines on safe media reporting [12,13]. We also suggested to journalists that they include in their reports information about organizations (such as the Samaritans) offering support to those with suicidal thoughts. With Professor Kapur's consent, we contacted these organizations ahead of the paper's publication to let them know about the study's findings and the likely upcoming media attention.

By the time this current editorial is published, we will be able to assess how the media covered Kapur and colleagues' study, and we will summarize this coverage in a blog post. Monitoring the media, and highlighting the best and worst quality coverage, is an important tool for improving media portrayal of suicide [5,14]. There are high stakes in getting this portrayal right. Reporters have a choice about how they frame stories about mental illness—Australia's Media Monitoring Project states that this choice has “serious ramifications for the ways in which audiences may interpret news and information about suicide, and mental health and illness.” If the right choices are made, says the project “they can help to destigmatise mental illness in our community and improve the lives of many people with mental illnesses” [16].
